# Indentation Behavior and Mechanical Properties of Tungsten/Chromium co-Doped Bismuth Titanate Ceramics Sintered at Different Temperatures

**DOI:** 10.3390/ma11040503

**Published:** 2018-03-27

**Authors:** Shaoxiong Xie, Jiageng Xu, Yu Chen, Zhi Tan, Rui Nie, Qingyuan Wang, Jianguo Zhu

**Affiliations:** 1 College of Architecture and Environment, Sichuan University, Chengdu 610065, China; xsxdyx@126.com; 2 School of Architecture and Civil Engineering, Chengdu University, Chengdu 610106, China; xxujiageng@163.com; 3 College of Materials Science and Engineering, Sichuan University, Chengdu 610065, China; tanzhi0838@163.com (Z.T.); nierui129@163.com (R.N.); nic0400@scu.edu.cn (J.Z.); 4 School of Mechanical Engineering, Chengdu University, Chengdu 610106, China

**Keywords:** Bi_4_Ti_3_O_12_ ceramics, sintering temperature, crack propagation, mechanical properties, indentation behavior

## Abstract

A sort of tungsten/chromium(W/Cr) co-doped bismuth titanate (BIT) ceramics (Bi_4_Ti_2.95_W_0.05_O_12.05_ + 0.2 wt % Cr_2_O_3_, abbreviate to BTWC) are ordinarily sintered between 1050 and 1150 °C, and the indentation behavior and mechanical properties of ceramics sintered at different temperatures have been investigated by both nanoindentation and microindentation technology. Firstly, more or less Bi_2_Ti_2_O_7_ grains as the second phase were found in BTWC ceramics, and the grain size of ceramics increased with increase of sintering temperatures. A nanoindentation test for BTWC ceramics reveals that the testing hardness of ceramics decreased with increase of sintering temperatures, which could be explained by the Hall–Petch equation, and the true hardness could be calculated according to the pressure-state-response (PSR) model considering the indentation size effect, where the value of hardness depends on the magnitude of load. While, under the application of microsized Vickers, the sample sintered at a lower temperature (1050 °C) gained four linearly propagating cracks, however, they were observed to shorten in the sample sintered at a higher temperature (1125 °C). Moreover, both the crack deflection and the crack branching existed in the latter. The hardness and the fracture toughness of BTWC ceramics presented a contrary variational tendency with increase of sintering temperatures. A high sintering tends to get a lower hardness and a higher fracture toughness, which could be attributed to the easier plastic deformation and the stronger crack inhibition of coarse grains, respectively, as well as the toughening effect coming from the second phase.

## 1. Introduction

Bismuth layered structure ferroelectrics (BLSF), as a kind of deuterogenic perovskite compounds with a high Curie temperature, have become a competitive candidate for the sensitive materials of those piezoelectric/ferroelectric devices with high operating temperatures [1] in view of their interesting electromechanical-coupling behaviors and fatigue-free properties [2]. In the family of BLSFs, Bi_4_Ti_3_O_12_ (BIT) as a typical member has attracted great interest during the 1970s because of its high Curie temperatures of ~675 °C and large spontaneous polarization of ~50 μC/cm^2^ along the *a*-axis. BIT has been reported to have considerable potential for application in some high-temperature (300 °C~400 °C) piezoelectric transducers after its high electrical conductivity were significantly decreased by Nb-doping [3]. 

In most piezoelectric sensors and actuators, ferroelectric ceramics are prone to fatigue due to cyclic electrical or mechanical loadings. The fatigue manifests its effect as a reduction in domain switching ability or mechanical strength and subsequent premature failure of devices. However, such mechanical properties of ferroelectric ceramics, which significantly influences the reliability of the devices. These often neglected because we used to pay more attention to their electrical properties, which are more relevant to the output of devices. Especially in some severe working environments involved with high temperature, strong coupling and high frequency, etc. [4,5], some complicated mechanical behavior of ferroelectric ceramics including fatigue crack propagation, creep deformation, and brittle–ductile transition have to be considered in the structural design of devices for the guarantee of reliability. To characterize the mechanical behavior of ferroelectric ceramics is of paramount importance in understanding their in-service failure mechanisms based on the knowledge that the sharp indenter has considerable potential as a microprobe for quantitatively characterizing mechanical properties. The indentation technology has been widely used in the last three decades for measuring the mechanical properties of small-scale materials such as electrical ceramics [6,7]. On the other hand, it is well known that the sintering temperatures plays an important role in the microstructural development of ceramics, further influencing its macroscopic mechanical properties [8,9,10,11]. 

Recently, W/Cr co-doped Bi_4_Ti_3_O_12_ ceramics were identified to have a low electric conductivity and a high piezoelectric constant [12,13]. However, there is hardly any report referring to the influence of sintering temperatures on the mechanical properties of this material. In this paper, a sort of W/Cr co-doped Bi_4_Ti_3_O_12_ ceramics with the optimal chemical composition were synthesized by a traditional ceramic process. We investigated the microstructural evolution of ceramics with the sintering temperatures, revealing the correlation between the deformation mechanism and microstructures of ceramics by two mechanical testing including nanoindentation and Vickers indentation. 

## 2. Experiment

### 2.1. Preparation of Ceramics

A sort of W/Cr co-doped Bi_4_Ti_3_O_12_ ceramics with a chemical formula of Bi_4_Ti_2.95_W_0.05_O_12.05_ + 0.2 wt % Cr_2_O_3_ (abbreviated as BTWC), were fabricated by two steps using the conventional solid-state reaction technique. First of all, reagent-grade oxide powders: Bi_2_O_3_ (99.999%), TiO_2_ (98%) and WO_3_ (99%) (Sinopharm Chemmical Reagent Co., Ltd., Shanghai, China) were weighed in the stoichiometric amounts (Bi_4_Ti_2.95_W_0.05_O_12.05_) of the ceramics. These raw materials were mixed by planetary ball mill using ethanol as solvent and zirconia as grinding balls for 24 h. This homogeneous mixture was calcined at 850 °C for 4 h to synthesize the compound of Bi_4_Ti_2.95_W_0.05_O_12.05_ after drying. Secondly, 0.2 wt % of Cr_2_O_3_ (99%) was added into the calcined powders and then mixed with them in the same method. The dried powders were granulated with polyvinyl alcohol (PVA, 8%). And then, the powders were compacted into discs with a diameter of 10 mm and a thickness of 1 mm under an isostatic stress of 150 MPa. After PVA was burned out at 450 °C, these discs were sintered at a temperature range of 1050–1150 °C for 4 h in a sealed alumina crucible to get BTWC ceramics. 

### 2.2. Characterization of Ceramics

#### 2.2.1. Microstructural Characteristics

The actual density of ceramics was measured by the Archimedes method. The phase structures of ceramics were determined by an X-ray diffractometer (XRD, DX2700, Dandong, China) using Cu-Kα radiation (λ = 1.5418 Å) at room temperature. The microstructural morphology of the ceramics was observed by scanning electron microscopy (SEM, JSM-610LV, JEOL, Tokyo, Japan) focusing on their natural surfaces. The average grain size was obtained by the linear intercept method from the SEM images.

#### 2.2.2. Nanoindentation Test

A nanoindentation testing system (Hysitron Triboscope, Hysitron, Eden Prairie, MN, USA) conducted by a Berkovich indenter was employed to investigate the elastic and plastic properties of the ceramics. Firstly, the surfaces of ceramics were finely polished using diamond pastes. In all tests, both the loading rate and the unloading rate were maintained at 0.5 mN/s for each peak load (50 mN, 100 mN, 150 mN and 200 mN). Forces and displacements were synchronously recorded to obtain the load-depth curves. The hardness (*H*) and other parameters were determined according to the Oliver–Pharr method as following formulas [14],

(1) $$H=\frac{P}{A}$$

(2) $$A=24.56h_{c}^{2}$$

(3) $$h_{c}=h-\varepsilon \frac{P}{S}$$

(4) $$E_{r}=\frac{\sqrt{\pi }}{2}\times \frac{S}{\sqrt{A}}$$

(5) $$\frac{1}{E_{r}}=\frac{1-v^{2}}{E_{}}+\frac{1-v_{i}{}^{2}}{E_{i}}$$

where *P* is the peak indentation load; *A* is the project area of the hardness impression; *h*_c_ is the contact depth deduced from the resultant load-displacement curves; *h* is the maximum depth of penetration; *ε* is the indenter geometry constant. For the conical indenter, *ε* has an empirical value of 0.75; *S* is the stiffness determined by the upper portion of the unloading data; *E*_r_ is the reduced modulus; *E* and *ν* are the elastic properties of the samples, and Young’s modulus and Poisson’s ratio of the diamond indenter are *E*_i_ = 1440 GPa and *ν*_i_ = 0.07 [15]. 

#### 2.2.3. Vickers Indentation Test

A Vickers diamond indenter (AKASHI, AVK-A, Tokyo Japan) was used for measuring the hardness and fracture toughness of ceramics with a polished surface. The indentation load of 19.6 N was applied and the holding time was 15 s. The hardness and fracture toughness were determined by the indentation fracture technique and the geometry patterns of the indentation and cracks were observed by SEM. The values of hardness (*H*) was measure according to the ASTMC 1327-99 and fracture toughness (*K_IC_*) was proposed by Anstis [16], which were calculated by following formulas,

(6) $$H=1.8544\frac{P}{d^{2}}$$

(7) $$K_{IC}=0.016{\left(\frac{E}{H}\right)}^{1/2}\left(\frac{P}{C^{3/2}}\right)$$

where, the value of *P* is 19.6 N, *d* is the average length of diagonal under indentation, *E* is the Young’s modulus obtained from the nanoindentation test and *C* is the length of crack measured from the center of the indentation.

## 3. Result and Discussion

**Figure 1:**
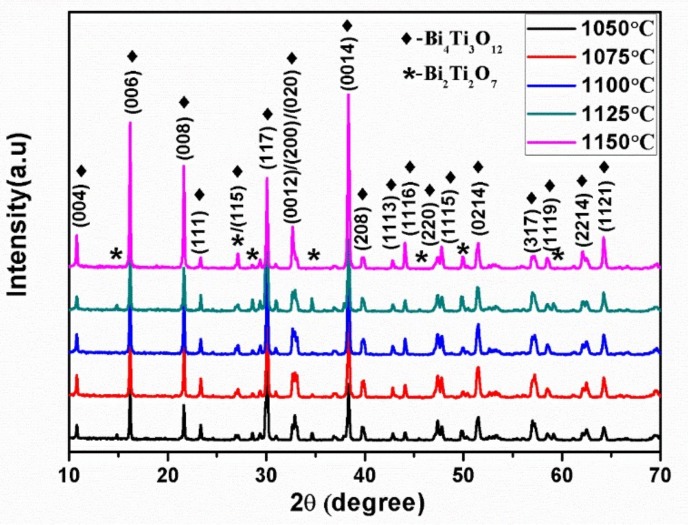
wt %XRD patterns of BTWC ceramics sintered at different temperatures.

Figure 1 shows the XRD patterns of the BTWC ceramics sintered at different temperatures. The diffraction peaks marked by rhombs were well-indexed as Bi_4_Ti_3_O_12_ with an orthorhombic structure and a space group of *B*2*cb* (41) in the light of JCPDS card # 72-1019. It suggests that all WO_3_ and Cr_2_O_3_ doped were successfully diffused into the crystal lattice of Bi_4_Ti_3_O_12_, forming a solid solution with the matrix. Considering the preparing process of BTWC ceramics, WO_3_ of 5 mol % was firstly added into the raw material according to the stoichiometric composition of Bi_4_Ti_2.95_W_0.05_O_12.05_ (BITW), thus these W^6+^ introduced will occupy those Ti^4+^ vacancies designed in the starting composition of powders. While Cr_2_O_3_ of 0.2 wt % as a fully redundant component were then added into the as-calcined BITW powders, thus Cr^3+^ can only substitute Ti^4+^ in the later sintering process of ceramics [17]. In the [TiO_6_] octahedron of Bi_4_Ti_3_O_12_, the substitution of W^6+^ and Cr^3+^ for Ti^4+^ could be attributed to their similar ionic radius (W^6+^: 0.600 Å, Cr^3+^: 0.615 Å and Ti^4+^: 0.605 Å) and matching coordination number based on the theory of crystal chemistry. However, a secondary phase (asterisked) was detected in all samples, which was identified as Bi_2_Ti_2_O_7_ with a cubic structure according to JCPDS card # 32-0118. In the preparation process of BTWC ceramics, Bi_2_Ti_2_O_7_ is prone to form in case of an initial Ti/Bi ratio higher than 3/4 during calcining and may also be resulted from the decomposition of Bi_4_Ti_3_O_12_ during sintering [18]. According to the intensity ratio between the impurity phase and the total phases [19], the phase content of Bi_2_Ti_2_O_7_ in each sample can be figured out as follows: 6.63% (1050 °C), 2.52% (1075 °C), 2.46% (1100 °C), 10.18% (1125 °C) and 3.27% (1150 °C), respectively. The sample sintered at 1125 °C seems to contain more Bi_2_Ti_2_O_7_ than the others, which may be caused by the heavy decomposition of Bi_4_Ti_3_O_12_ at this sintering temperature.

**Figure 2:**
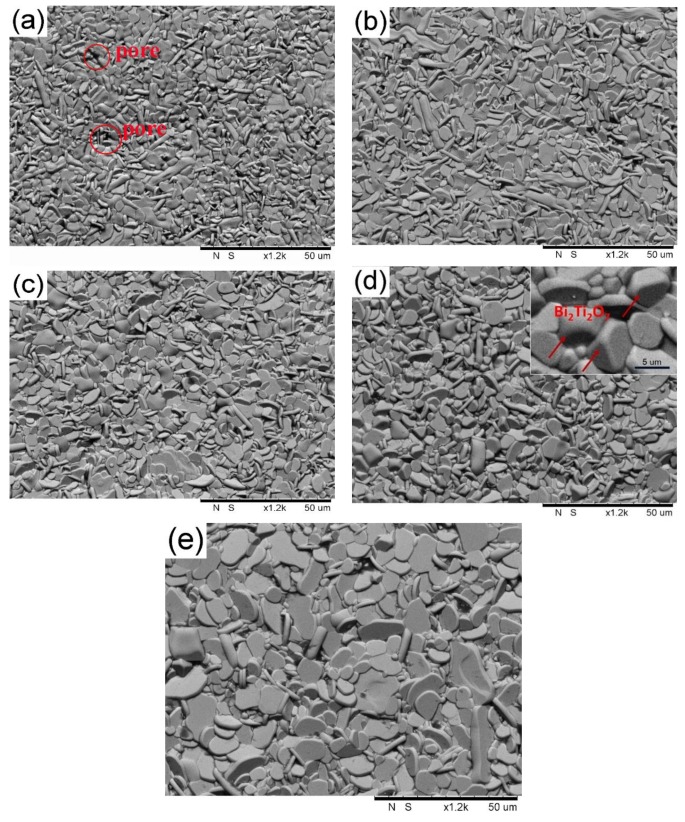
SEM photos on the natural surfaces of BTWC ceramics sintered at different temperatures: (**a**) 1050 °C; (**b**) 1075 °C; (**c**) 1100 °C; (**d**) 1125 °C; (**e**) 1150 °C.

Figure 2 shows the microstructures on the natural surfaces of BTWC ceramics sintered at different temperatures. It can be found that all these samples were mainly composed of the plate-like grains with random orientation. This special grain morphology with a high aspect ratio is contributed by a higher grain growth rate along the *a*-*b* plane of the crystal [9], which is essentially related to a lower interfacial energy in this crystallographic plane [17]. With the increase of sintering temperatures from 1050 to 1150 °C, the average length of plate-like grains slowly increases from 3.2, 4.1, 5.3 to 7.6 μm in the prophase, but soars to 16.1 μm in the end. The sample sintered at 1150 °C (Figure 2e) presents an extreme grain growth, which may be attributed to the formation of liquid phase accelerating the grain boundary diffusion at the higher sintering temperature. In addition, it can be seen from Figure 2a that some pores could be observed in the sample sintered at 1050 °C. The porosity could reflect the densification effect of ceramics, and some appropriate donor dopants are suggested to favor the densification process of Bi_4_Ti_3_O_12_ ceramics by a solute drag mechanism [20]. Also, the liquid phase occurring in the grain boundary during sintering may also promote the densification process of ceramics [21]. On the other hand, one can also see that some small polyhedral grains were mingled with these big plate-like grains. These heteroid grains are indexed as Bi_2_Ti_2_O_7_ impurity, and their apparent amount basically agree with their phase content for each sample. Here, the sample sintered at 1125 °C seems to contain more impurity as shown in Figure 2d and the inserted figure, a normal growth of Bi_4_Ti_3_O_12_ grains may be depressed by the competitive growth of Bi_2_Ti_2_O_7_ grains [22]. The microstructural evolution of BTWC ceramics with the sintering temperatures are summarized in Table 1. The relative density of all the samples exceed 93%, especially, the sample sintered at 1150 °C obtained a high density of 97.83%, which may be profited from both its intrinsic less impurity phase and extrinsic more liquid phase during sintering.

**Figure 3:**
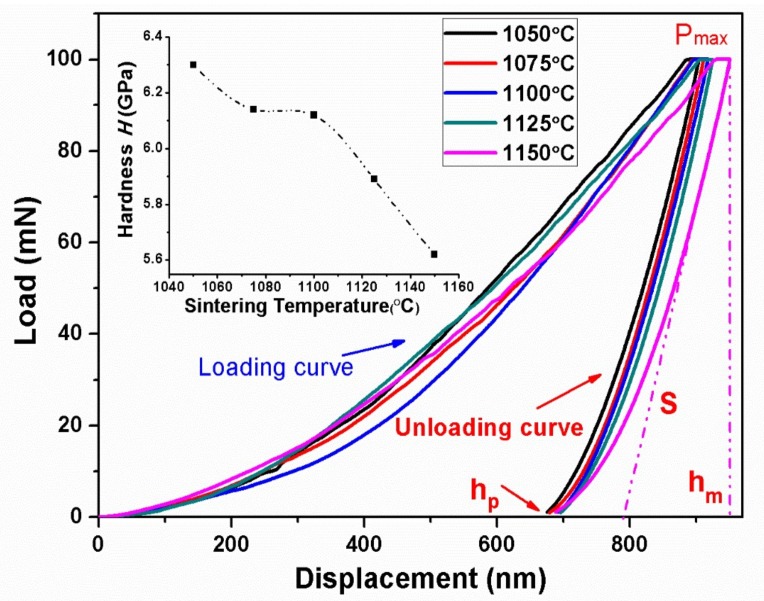
Load-displacement curves of BTWC ceramics sintered at different temperatures in the nanoindentation test.

Figure 3 shows the typical load-displacement curves of BTWC ceramics sintered at different temperatures, which are derived from the nanoindentation test. A peak load of 100 mN was applied to these samples. It can be seen that both the loading curves and the unloading curves were successive and smooth for all samples, that is to say that there is no local brittle fracture occurring in the whole deformation process of BTWC ceramics, thus a fine ductility could be considered for them when subjected to the applied load. In addition, the maximal displacement of samples presents a slight increase following the increase in sintering temperatures, and a significant increase was observed at 1150 °C, which indicates that the change of sintering temperature leads to the evolution of mechanical behavior for BTWC ceramics. In fact, a different elastic–plastic deformation mechanism was concealed under the applied stress. Here, the inserted map in Figure 3 describes the hardness of BTWC ceramics as a function of their sintering temperatures. As can be seen that the hardness value showed a continuous downtrend with the increase of sintering temperatures. This result can be explained by the classical Hall–Petch relation as follows [23],

(8) $$H=H_{0}+kd^{-1/2}$$

where, *H*_0_ and *k* are material constants, *d* is the grain size of materials. It shows that a higher hardness is usually existing in the fine-grained materials. For BTWC ceramics, it has been identified by SEM that the grain size increase with the increase of sintering temperatures, which determines the decrease of hardness by the Hall–Petch relation. Therefore, the fine-grained ceramics are prone to have a harder plastic deformation because of its higher hardness.

Here, some mechanical properties of BTWC ceramics obtained by the nanoindentation test are summarized in Table 2. The Young’s modulus (*E*) of solid materials indicates the ability to resist the elastic deformation. As for ceramics, it is considered to have some connection with the strength of ionic bonds and covalent bonds, the porosity and the second grains [24]. The highest Young’s modulus of 98.57 GPa is obtained by the sample sintered at 1125 °C, which has the highest phase content of Bi_2_Ti_2_O_7_. It has been reported that Bi_2_Ti_2_O_7_ has a smaller deformation than Bi_4_Ti_3_O_12_ when they are subjected to external force, due to the lower axial ratio of its cubic cell [25]. On the other hand, all the maximum depth (*h*), contact depth (*h*_c_) and residual deformation depth (*h*_p_) roughly present an uptrend with the increase of sintering temperatures. The sample sintered at 1050 °C responds a smaller deformation (both elastic and plastic) to the applied stress compared with the others, which could be ascribed to the fact that the fine-grained materials may bring about additional obstacles for dislocation movement in the adjacent grains. However, the residual depth caused by the plastic deformation is abundant for BTWC ceramics. The proportion of residual deformation depth in the total indentation penetration depth exceeds 50%. Thus, the plastic deformation dominates the total deformation of BTWC ceramics. It suggests that BTWC ceramics have a stronger ability to resist the brittle fracture. 

**Figure 4:**
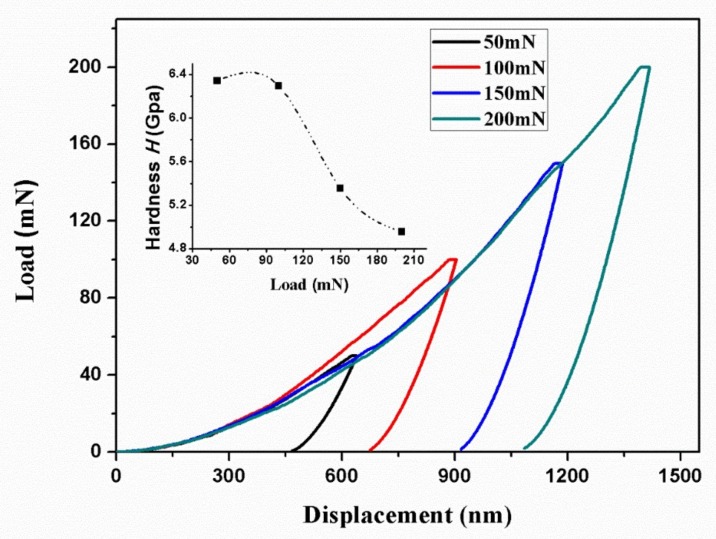
Load-displacement curves at different peak loads for BTWC ceramics sintered at 1050 °C.

It is worth mentioning that the apparent hardness of materials tested directly by the nanoindentation is different with its true hardness, which is due to the indentation size effect resulted from different loads [26,27]. Li and Bradt proposed a proportional specimen resistance (PSR) model as an adequate approach to explain the nanoindentation data of ceramics [28]. In this model, the relationship between the effective indentation load and the indentation dimension can be described by the following formula:

(9) $$\frac{P_{\max}}{h_{c}}=\alpha_{1}+\alpha_{2}h_{c}$$

where, *P*_max_ is the maximum applied load and *h*_c_ is the corresponding indentation contact depth; *a*_1_ and *a*_2_ are two constants of the test material, which is determined by its elastic and plastic properties, respectively. In addition, according to the energy balance consideration proposed by Quinn [29], *a*_1_ and *a*_2_ are affiliated with the energies dissipated in the process of producing a new surface of an unit area and forming the irreversible deformation of an unit volume, respectively. For a nanoindentation test with a Berkovich indenter, both *a*_1_ and *a*_2_ are a measure parameters of the true hardness, *H*_0_, which can be determined directly by the following formulas:

(10) $$H_{01}=\frac{P_{\max}-\alpha_{1}h_{c}}{24.5h_{c}^{2}}$$

(11) $$H_{02}=\frac{\alpha_{2}}{24.5}$$

where, *H*_01_ and *H*_02_ are the true hardness related to the *a*_1_ and *a*_2_, respectively. *a*_1_ and *a*_2_ can be derived from the plot of *P*_max/_*h*_c_ versus *h*_c_ according to Equation (9). To get the plots of *P*_max_/*h*_c_ versus *h*_c_, several peak loads from 50, 100, 150 to 200 mN were applied to BTWC ceramics. Here, taking the sample sintered at 1050 °C to show the load-displacement curves at different peak loads (Figure 4). The others have a similar result.

**Figure 5:**
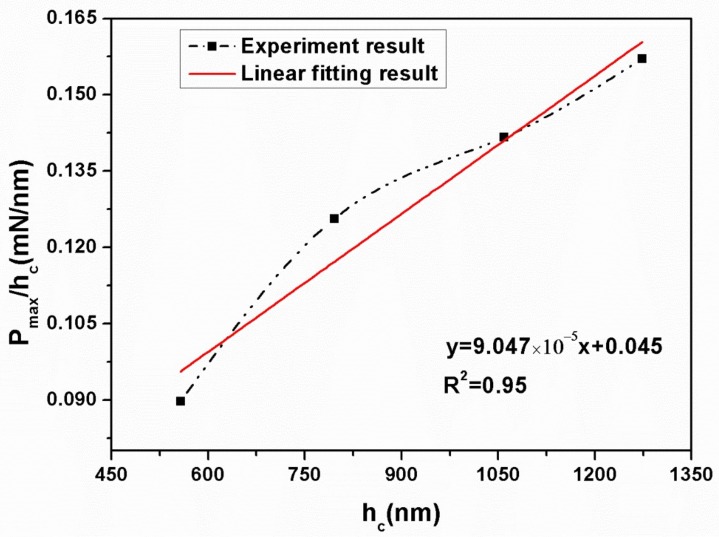
Plots of *P*_max_/*h*_c_ versus *h*_c_ and the linear fitting result.

As can be seen from Figure 4, both the maximum penetration depth and the residual depth are observed to increase with increasing peak loads. However, the hardness gained by the nanoindentation test presented a downtrend with increasing peak loads as shown in the inserted map. The hardness gains the lowest value of 4.96 GPa at the peak load of 200 mN, while the highest value of 6.34 GPa at 50 mN. It shows that the indentation size effect is significant and the hardness is dependent on the peak load. Based on Equation (9), the *P*_max_/*h*_c_ versus *h*_c_ curve was depicted in Figure 5, and the fitting result (R^2^ = 0.95) gives out the value of *a*_1_ and *a*_2_ as 0.045 mN/nm and 9.047 × 10^−5^ nN/nm^2^, respectively. And then, the value of true hardness (*H*_01_ and *H*_02_) at different peak loads can be evaluated according to Equations (10) and (11).

**Figure 6:**
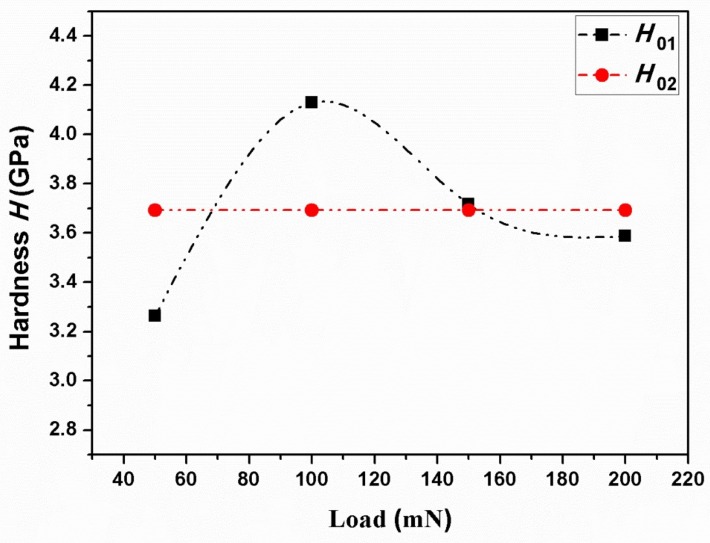
*H*_01_ and *H*_02_ of the sample sintered at 1050 °C as a function of peak loads.

**Figure 7:**
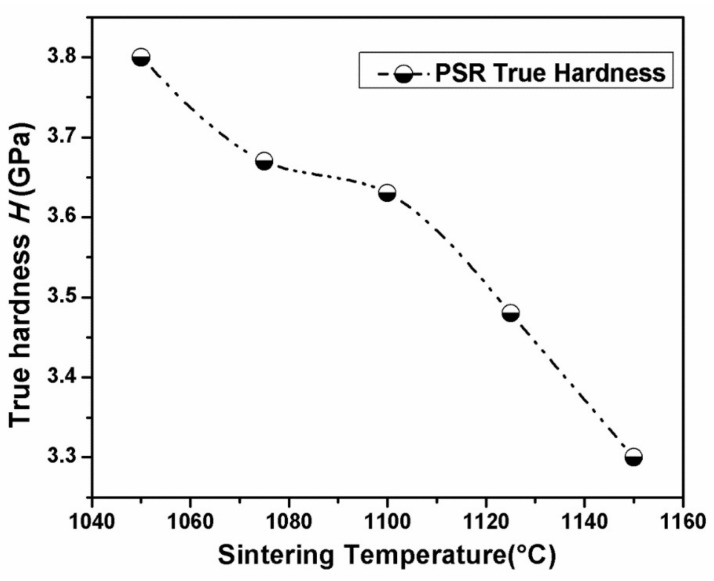
PSR true hardness of BTWC ceramics sintered at different temperatures.

Based on the PSR model, *H*_01_ and *H*_02_ as a function of the peak load are shown in Figure 6. It can be seen that *H*_01_ fluctuates around *H*_02_ with increasing peak loads. Their central value are located at ~3.8 GPa, which are much lower than the testing value of hardness (*H* = 6.3 GPa). This result proves the size effect existing in the nanoindentation test for hardness. In addition, the PSR true hardness of BTWC ceramics sintered at different temperatures were shown in Figure 7.

As we know, although the nanoindentation technique fits for investigating the elastic–plastic transition behavior of ceramics at the scale of nanometers, cracks around the indentation can’t be observed easily, since a small amount of applied stress is unlikely to produce cracks. Therefore, it is unavailable to test the fracture toughness for ceramics. On the other hand, the Vickers based on a conventional microindentation technique could create both indentations and cracks through a stronger stress field applied for brittle materials. Since such cracks and indentations can be clearly observed by SEM, this method is usually employed to test the fracture toughness for brittle materials including ceramics and glasses, etc. based on the theory of fracture mechanics.

**Figure 8:**
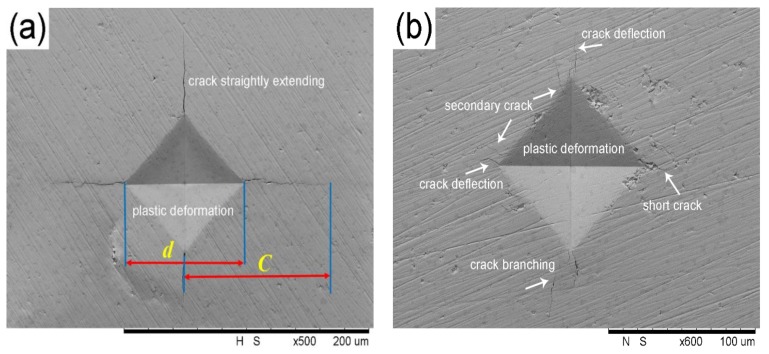
Patterns of Vickers indentation and resulting cracks derived from BTWC ceramic sintered at (**a**) 1050 °C and (**b**) 1125 °C.

The typical patterns of the indentation and crack produced by the Vickers are shown for BTWC ceramics in Figure 8. In Figure 8a, it can be seen that a symmetric rhombic indentation was induced by the Vickers diamond indenter on the surface of ceramics, and four cracks straightly propagated along the diagonal direction of rhombic indentation. In the mechanical model of sharp indenter applied for brittle materials, the stress field contributes two superposable components including an elastic (reversible) part and a residual (irreversible) part to the net driving force on the crack system [30]. At the indentation surface, the elastic component is compressive, while the residual component is tensile. Thus the radial cracks grow to their final lengths as the indenter is unloaded, i.e., as the restraining elastic field is removed. Therefore, the area within the rhombic indentation is regarded as the plastic deformation zone formed by the application of sharp indenter, while the crack area outside of the rhombic indentation is considered as the elastic deformation zone used for releasing partial strain energy. Thus the characteristics of cracks can be related to the fracture characteristic of materials. For the sample sintered at 1050 °C, four cracks are observed to linearly extend for a long distance and then gradually disappear in Figure 8a, which is known as a normal model of crack propagation in piezoceramics subjected to a small indentation load. However, this normal crack propagation has changed in the sample sintered at 1125 °C as shown in Figure 8b. Firstly, all cracks are shorter and thinner; secondly, the main crack initiates accompanied with a secondary crack; thirdly, some cracks deflect from its initial propagation direction after extending a short distance, and even a few cracks are tardily split into some slight branches following the main crack. Here, the crack shortening, the secondary crack, the crack defection or the crack branching all them could be attributed to the microstructural aspects (including structural inhomogeneity of matrix, larger aspect ratio of grains and microcracks pre-existing in matrix, etc.) of ceramics according to the description in reference [31]. In addition, it has been identified by SEM and XRD that this sample contains a large number of Bi_2_Ti_2_O_7_ second grains, which tend to cause many residual stress in the sintering process of ceramics because of the thermal expansion mismatch between two different phases. Hence, there may be some microcracks induced by the residual stress existing in the matrix, which are considered to initiate second cracks around main cracks by their slow propagation under the stress field. Moreover, they can also weaken the stress field around crack tips (causing the crack defection or branching), or depress the driving force of crack propagation (causing the crack shortening). 

**Figure 9:**
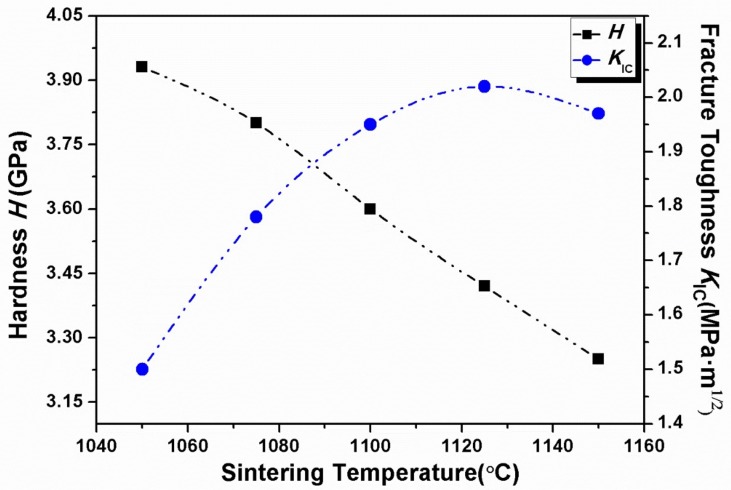
Hardness and fracture toughness of BTWC ceramics sintered at different temperatures.

Figure 9 displays *H* and *K_IC_* of BTWC ceramics as a function of their sintering temperatures beneath the Vickers diamond indenter, and their values including the average length of diagonal (*d*) and crack length (*C*) are shown in Table 3. It can be seen that the hardness decreased gradually with increasing the sintering temperatures from 1050 to 1150 °C, which is just opposite to the uptrend of grain size with the sintering temperatures (Table 1). Therefore, this result also agrees with the Hall-Petch equation which indicates the inverse relation between the hardness and the grain size for brittle ceramics. Moreover, for each sample, its PSR true hardness are basically equal to the Vickers hardness as observed from Figure 7 and Figure 9. In fact, the indentation size effect also exists in Vickers test. Vickers hardness-load curve of brittle ceramics usually presents a hardness-platform with increasing applied loads [29], and the level value of hardness is identified as the true hardness of materials. In this experiment, the indentation load of 19.8 N could be considered to fit for BTWC ceramics in terms of such a fine morphology of cracks shown in Figure 8, and it corresponds to the applied load for the hardness approaching its constant value, which is similar with the load dependence of the PSR true hardness in the nanoindentation test.

On contrary, the fracture toughness of BTWC ceramics exhibited an approximate uptrend with increasing sintering temperatures. In the case of a higher sintering temperature, the resulting coarse grains can absorb much of the energy of crack propagation with the help of the crack deflection or branching mechanism [21]. As a result, the fracture toughness of materials tends to increase with increase of grain size. However, the highest fracture toughness of 2.02 MPa·m^1/2^ is given to the sample sintered at 1125 °C rather than the sample sintered at 1150 °C, which has the largest grain size. Maybe, we can understand this result based on the fact as follows. Firstly, this sample contains more Bi_2_Ti_2_O_7_ than the others. It has been reported that the second phase with a higher thermal expansion coefficient will induce a compressive stress field in the matrix to absorb the fracture energy, blunt crack tips and shield crack propagation leading to a higher fracture toughness [32]. Besides, the second phase also induces microcracks to the matrix, which are considered as one type of toughening mechanism for ceramics. Therefore, the sample sintered at 1125 °C gains a larger fracture toughness than the others, which could be ascribed to its higher sintering temperatureand numerous second phase grains.

## 4. Conclusions

Indentation behavior and mechanical properties of W/Cr co-doped Bi_4_Ti_3_O_12_ ceramics (BTWC) sintered at different temperatures were investigated. XRD revealed that Bi_4_Ti_3_O_12_ as the major phase and Bi_2_Ti_2_O_7_ as the second phase coexisted in BTWC ceramics, and the phase content of Bi_2_Ti_2_O_7_ varied with sintering temperatures. SEM demonstrated that BTWC ceramics were composed of the plate-like grains with random orientation, and the grain size increased with increase of sintering temperatures. In the nanoindentation test, the hardness value (5.62 GPa~6.3 GPa) of BTWC ceramics was found to decrease with increase of sintering temperatures, which could be explained by the Hall–Petch equation, and their true hardness (3.3 GPa~3.8 GPa) could be calculated by the PSR model considering the indentation size effect. In the Vickers indentation test, these cracks produced tended to be shortened in the sample sintered at a higher temperature (1125 °C), as well as presented deflection and branching. The hardness and the fracture toughness of BTWC ceramics had a contrary variational tendency with sintering temperatures.

## Figures and Tables

**Table 1:** Microstructural evolution of BTWC ceramics with the sintering temperatures.

Sintering Temperature	1050 °C	1075 °C	1100 °C	1125 °C	1150 °C
Phase content of Bi_2_Ti_2_O_7_ (%)	6.63	2.52	2.46	10.18	3.27
Average grain size of Bi_4_Ti_3_O_12_ (μm)	3.2	4.3	5.1	7.6	16.1
Relative density of ceramics (%)	95.42	97.09	95.36	96.71	97.83

**Table 2:** Mechanical properties of BTWC ceramics determined by the nanoindentation test.

Sintering Temperature	*H* (GPa)	*E* (GPa)	*h* (nm)	*h*_c_ (nm)	*h*_p_ (nm)
1050 °C	6.30	96.07	904.51	795.71	644.07
1075 °C	6.14	98.13	911.15	805.44	663.29
1100 °C	6.12	93.49	918.08	807.19	675.80
1125 °C	5.89	98.57	924.91	822.58	662.88
1150 °C	5.62	90.00	951.34	842.80	682.11

**Table 3:** The values of parameters obtained by Vickers indentation test.

Sintering Temperature	*d* (μm)	*H* (Gpa)	*P* (N)	*C* (μm)	*K* (MPa·m^1/2^)
1050 °C	96.19	3.93	19.6	102.16	1.50
1075 °C	97.83	3.80	19.6	92.86	1.78
1100 °C	100.59	3.59	19.6	87.69	1.95
1125 °C	103.15	3.42	19.6	88.74	2.02
1150 °C	105.83	3.25	19.6	88.83	1.97
